# HER2 overexpression and amplification as a potential therapeutic target in colorectal cancer: analysis of 3256 patients enrolled in the QUASAR, FOCUS and PICCOLO colorectal cancer trials

**DOI:** 10.1002/path.4679

**Published:** 2016-01-29

**Authors:** Susan D Richman, Katie Southward, Philip Chambers, Debra Cross, Jennifer Barrett, Gemma Hemmings, Morag Taylor, Henry Wood, Gordon Hutchins, Joseph M Foster, Assa Oumie, Karen G Spink, Sarah R Brown, Marc Jones, David Kerr, Kelly Handley, Richard Gray, Matthew Seymour, Philip Quirke

**Affiliations:** ^1^ Section of Pathology and Tumour BiologyLeeds Institute of Cancer and Pathology, University of LeedsUK; ^2^ Histopathology and Molecular PathologySt James University HospitalLeedsUK; ^3^ Section of Epidemiology and BiostatisticsLeeds Institute of Cancer and Pathology, University of LeedsUK; ^4^ Affymetrix UK LtdHigh WycombeUK; ^5^ Clinical Trials Research UnitUniversity of LeedsLeedsUK; ^6^ Cancer MedicineUniversity of OxfordUK; ^7^ Birmingham Clinical Trials UnitUniversity of BirminghamUK; ^8^ Clinical Trials Service Unit and Epidemiology Studies UnitUniversity of OxfordUK; ^9^ Section of OncologyLeeds Institute of Cancer and Pathology, University of LeedsUK

**Keywords:** HER2, colorectal cancer, amplification, overexpression, copy number variation

## Abstract

HER2 overexpression/amplification is linked to trastuzumab response in breast/gastric cancers. One suggested anti‐EGFR resistance mechanism in colorectal cancer (CRC) is aberrant MEK–AKT pathway activation through HER2 up‐regulation. We assessed HER2‐amplification/overexpression in stage II–III and IV CRC patients, assessing relationships to KRAS/BRAF and outcome. Pathological material was obtained from 1914 patients in the QUASAR stage II–III trial and 1342 patients in stage IV trials (FOCUS and PICCOLO). Tissue microarrays were created for HER2 immunohistochemistry. HER2‐amplification was assessed using FISH and copy number variation. KRAS/BRAF mutation status was assessed by pyrosequencing. Progression‐free survival (PFS) and overall survival (OS) data were obtained for FOCUS/PICCOLO and recurrence and mortality for QUASAR; 29/1342 (2.2%) stage IV and 25/1914 (1.3%) stage II–III tumours showed HER2 protein overexpression. Of the HER2‐overexpressing cases, 27/28 (96.4%) stage IV tumours and 20/24 (83.3%) stage II–III tumours demonstrated HER2 amplification by FISH; 41/47 (87.2%) also showed copy number gains. HER2‐overexpression was associated with KRAS/BRAF wild‐type (WT) status at all stages: in 5.2% WT versus 1.0% mutated tumours (p < 0.0001) in stage IV and 2.1% versus 0.2% in stage II–III tumours (p = 0.01), respectively. HER2 was not associated with OS or PFS. At stage II–III, there was no significant correlation between HER2 overexpression and 5FU/FA response. A higher proportion of HER2‐overexpressing cases experienced recurrence, but the difference was not significant. HER2‐amplification/overexpression is identifiable by immunohistochemistry, occurring infrequently in stage II–III CRC, rising in stage IV and further in KRAS/BRAF WT tumours. The value of HER2‐targeted therapy in patients with HER2‐amplified CRC must be tested in a clinical trial. © 2015 The Authors. Journal of Pathology published by John Wiley & Sons Ltd on behalf of Pathological Society of Great Britain and Ireland.

## Introduction

Colorectal cancer (CRC) is one of the leading causes of cancer‐related deaths, with 1.36 million new cases and > 0.5 million deaths/year worldwide 1. In stage IV disease, cytotoxic agents such as fluorouracil, irinotecan and oxaliplatin have led to improved survival. Further improvements in outcomes have been seen with the introduction of targeted therapies. These include the use of monoclonal antibodies against the epidermal growth factor receptor (EGFR) in patients with *KRAS* wild‐type (WT) tumours, although only a minority of patients respond to this approach.

The HER family of tyrosine kinase receptors consists of EGFR, HER2 (ErbB2), HER3 and HER4. They are responsible for cell survival and proliferation via signalling through the RAS–RAF–ERK and PI3K–PTEN–AKT pathways 2. HER2 has been extensively studied in breast cancer, where gene amplification and overexpression of the protein is seen in approximately 20% cases and is associated with increased risk of recurrence and a poorer prognosis 3, 4, 5. Treatment of these patients with the anti‐HER2 monoclonal antibody trastuzumab (Herceptin) has led to improved survival in metastatic breast cancer 6.

HER2 overexpression has also been identified in other cancers, such as gastric cancer 7. FDA approval was given for the use of trastuzumab in *HER2*‐positive metastatic gastric cancers following the results of the ToGA trial, where median overall survival was 13.8 months in patients given trastuzumab with chemotherapy, compared to 11.1 months in those patients given chemotherapy alone 8. Several studies have assessed HER2 overexpression in CRC, with some reporting membranous expression, varying in the range 2.1–11% in 9, 10, 11, 12, 13, 14, 15, and others reporting cytoplasmic overexpression in the range 47.4–68.5% 12, 16, 17.

Topoisomerase II*α* (TOP2A) alters the topology of DNA during transcription by causing transient double‐strand breaks. The *TOP2A* gene is located telomeric of *HER2* on chromosome 17q. It is co‐amplified with *HER2* in 35% of HER2‐positive breast cancers and is associated with response to anthracyclines and other agents, although evidence is mixed.

It is now accepted that in stage IV colorectal disease, *RAS* mutations confer resistance to anti‐EGFR therapy and *BRAF* mutations are an indicator of poor prognosis; however, even among patients with *RAS* WT tumours, only a minority respond. One suggested mechanism of drug resistance is aberrant signalling through the up‐regulation of other transmembrane receptors, such as HER2. Here we compared the frequency of HER2 protein overexpression between 1914 stage II–III CRC patients from the QUASAR clinical trial with 1342 patients from two CRC stage IV clinical trials, 888 from FOCUS and 454 from PICCOLO. HER2 protein expression was assessed in relation to *HER2* amplification, as determined by FISH, *KRAS* and *BRAF* mutations, recurrence rates (in QUASAR patients) and finally to survival outcomes. Cases showing overexpression were also assessed for whole‐genome copy number variation, *HER2* amplification and associated *TOP2A* amplification.

## Materials and methods

Patients were recruited to the QUASAR (QUick And Simple And Reliable) trial during May 1994–December 2003, and were randomly assigned to receive 5FU/folinic acid chemotherapy (*n* = 1622) or to observation (*n* = 1617). Trial details are reported elsewhere 18. Tissue microarrays (TMAs) were constructed containing all cases with sufficient tumour for three cores/array. Ethical approval was obtained from both the West Midlands Multi‐Centre Research Ethics Committee (JR/MT/MREC/02/7/56a) and the Northern and Yorkshire Research Ethics Committee (08/H0903/62) for pathological substudies in QUASAR (ISRCTN82375386).

The MRC CR08 FOCUS trial [Fluorouracil, Oxaliplatin and CPT11 (irinotecan): Use and Sequencing] ISRCTN79877428 recruited 2135 first‐line advanced colorectal cancer (aCRC) patients during 2000–2003, comparing strategies of sequential or combination cytotoxic therapy, without targeted agents 19. Prospective consent was obtained to retrieve stored pathological material for research (MREC/99/3/1). From the trial repository of 1700 samples, 888 samples contained sufficient tumour material to provide three tumour cores for TMA construction.

PICCOLO (Panitumumab, Irinotecan and Cyclosporin in COLOrectal cancer therapy) assessed second‐line aCRC therapies, including the EGFR‐targeted agent panitumumab. It recruited 1198 patients during 2006–2010 20, 21. Patient consent was obtained to retrieve surplus stored pathological material for research (REC/06/Q0906/38). TMAs were made containing 582 patient samples, whose resection samples contained sufficient tumour material to provide three tumour cores. Following TMA sectioning and staining, 128 had missing cores or insufficient tumour within cores, leaving 454 patients suitable for assessment here.

### HER2 immunohistochemistry

From each TMA, 5 µm sections were cut onto Superfrost Plus slides and dried overnight at 37°C. The slides were dewaxed, then incubated in 4% hydrogen peroxide solution in methanol for 20 min, prior to antigen retrieval in a pressure cooker for 2 min in unmasking solution (Vector Laboratories, Burlingame, CA, USA). The slides were incubated in a casein solution (Vector Laboratories) for 10 min and then incubated for 1 h at room temperature with the HER2 antibody (polyclonal rabbit anti‐human c‐erbB‐2 oncoprotein; cat no. A0485 from Dako, Ely, UK) at a dilution of 1:250. The slides were incubated for 30 min with secondary HRP‐conjugated anti‐rabbit (Envision+ System HRP‐labelled polymer, Dako), then developed using 3,3′‐diaminobenzidine (DAB). The slides were counterstained with Mayer's haematoxylin and lithium carbonate, dehydrated and mounted and then scanned using an Aperio scanner (Leica Microsystems, Milton Keynes, UK) for visualization and scoring. Tumour cores were deemed to show strong overexpression of HER2 if the majority of tumour cells displayed a ‘full basket weave’ pattern of membrane staining in > 90% of cell membranes; this level of expression is classified as 3+ using current consensus‐scoring guidelines 22. To confirm the TMA result and to assess the level of heterogeneity of HER2 expression, whole sections from the tumours with overexpression were also stained, according to the above protocol.

### HER2 fluorescence in situ hybridization (FISH)

FISH was carried out using the *HER2* Kit (Kreatech, Uckfield, UK) on 4 µm formalin‐fixed, paraffin‐embedded (FFPE) tissue sections from the tumours demonstrating HER2 overexpression by IHC, according to the manufacturer's instructions. Briefly, a rhodamine‐labelled *HER2* probe was used to identify the number of target *HER2* molecules, together with a FITC‐labelled control *CEP17* probe, to identify normal gene copy number. The ratio of *HER2* signal (red):*CEP17* (green) was calculated in order to determine the presence or absence of *HER2* amplification. The criterion for gene amplification was an average *HER2:CEP17* ratio of ≥ 2 across at least 50 distinct, non‐overlapping tumour nuclei.

### KRAS and BRAF pyrosequencing

For DNA extraction, the QIAamp DNA Micro kit (Qiagen, Manchester, UK) was used, employing the standard manufacturer's protocol. Primers for amplification and pyrosequencing were designed using Pyrosequencing Assay Design Software (Qiagen)*. KRAS* codons 12–13 were amplified in one PCR reaction; *KRAS* codon 61 and *BRAF* codon 600 were amplified separately. Full details of PCR reaction conditions have been published elsewhere 23. PCR products were pyrosequenced on a PyroMark ID system (Qiagen), following the manufacturer's protocols.

### Copy number variation analysis

Forty‐seven cases with overexpression/amplification of HER2 (25 stage II–III and 22 stage IV) were run on the OncoScan^®^ FFPE Assay Kit (Affymetrix) to identify copy number alterations, according to the manufacturer's instructions. Array fluorescence intensity (CEL) files were generated automatically from DAT files using Affymetrix^®^ GeneChip^®^ Command Console^®^ (AGCC) software v. 4.0. (Affymetrix). The CEL files were processed by OncoScan^®^ Console software to generate OSCHP, which were loaded into Nexus Express for OncoScan^®^ (BioDiscovery, Hawthorne, CA, USA) for analysis of clinically relevant copy number (CN) and loss of heterozygosity (LOH) events. In parallel, somatic mutations were visualised in a Somatic Mutation Viewer (Affymetrix) and cross‐referenced with the CN data for correlation with CN events.

### Statistical analysis

Statistical analysis was carried out using STATA v. 12. The proportion of tumours with strong HER2 overexpression was compared between *KRAS*/*BRAF* WT and mutated tumours, using Fisher's exact test. Cox's proportional hazards models were used to test for the effect of HER2 strongly positive versus negative tumours on recurrence rates, progression‐free survival (PFS) and overall survival (OS), separately for each trial. Combined estimates were obtained by fixed effects meta‐analysis, testing for heterogeneity, in the two stage IV trials.

## Results

### HER2 immunohistochemistry

**Figure 1:**
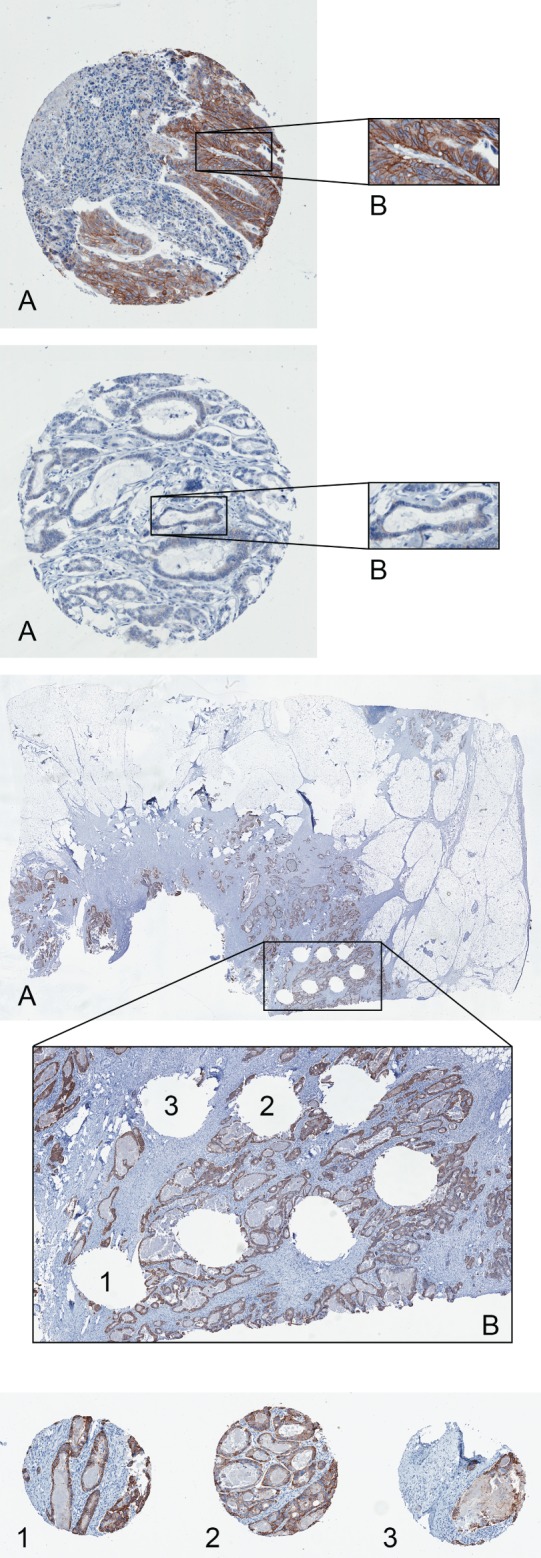
(A) Core from a colorectal tumour, demonstrating strong membranous overexpression of HER2; the cores were visually inspected at a native scanning resolution of ×20. (B) Core from a colorectal tumour, demonstrating weak membranous overexpression of HER2; the cores were visually inspected at a native scanning resolution of ×20. (C) Whole‐section staining was carried out to determine the homogeneity of HER2 overexpression across the tumour section; the location of each of the three cores that were stained on the TMA (1, 2 and 3) is also shown; the cores were visually inspected at a native scanning resolution of ×20

We observed strong membranous staining for HER2 (Figure 1A) in 25 of 1914 (1.3%) stage II–III tumours and 29 of 1342 (2.2%) stage IV tumours tested [18 of 888 (2.0%) in FOCUS; 11 of 454 (2.4%) in PICCOLO]. Weak membranous staining (Figure 1B) was observed in a further 53 of 1342 (3.9%) cases [44 of 888 (4.9%) in FOCUS, nine of 454 (2.0%) in PICCOLO tumours]. Corresponding whole‐tumour sections for the 29 stage IV tumours with strong membranous staining seen in the TMA cores were stained, and showed homogeneous overexpression across each tumour (Figure 1C).

### HER2 FISH analysis

**Figure 2:**
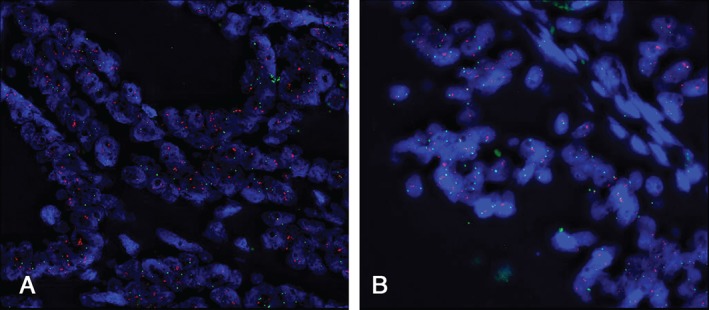
HER2 FISH analysis of two colorectal tumours, showing (A) amplification and (B) no amplification; red signals, HER2 probe; green signals, CEP17 probe; amplification is described as a HER2:CEP17 ratio of ≥ 2

Whole sections of the 54 cases with strong membranous staining by IHC were then subjected to HER2 FISH analysis; 24 of the 25 stage II–III tumours were successfully hybridized, with 20 of 24 (83.3%) showing HER2 amplification: 28 of 29 stage IV tumours were successfully hybridized, with 27 of 28 (96.4%) showing HER2 amplification (Figure 2); 10 of 53 stage IV tumours displaying weak membranous IHC staining were randomly selected for HER2 FISH analysis and none of these tumours showed HER2 amplification.

### HER2 copy number variation analysis by OncoScan

Forty‐seven cases where strong membranous staining of HER2 was observed were analysed for copy number variation of *HER2* and *TOP2A* using OncoScan: 41 of 47 (87.2%) showed amplification of *HER2*, two of 47 (4.3%) showed partial amplification of *HER2* and the remaining four showed no amplification of *HER2*; 17 of 47 (36.2%) showed co‐amplification of *HER2* and *TOP2A*. *HER2* amplification, as determined by FISH, correlated strongly with amplification, as determined by OncoScan analysis (Table 1).

**Table 1:** Correlation between amplification of HER2, as assessed by FISH, and amplification, as determined by OncoScan (n = 47)

	OncoScan		
	Amplification	Partial amplification	No amplification
FISH	*n* (%)	*n* (%)	*n* (%)
Amplification	38 (80.9)	n/a	2 (4.3)
No amplification	2 (4.3)	2 (4.3)	1 (2.1)
Assay failure	1 (2.1)	n/a	1 (2.1)

### HER2 and KRAS/BRAF mutation status

HER2 overexpression was strongly associated with *KRAS/BRAF* WT status in all stages of disease. In the QUASAR trial, HER2 overexpression was observed in 17 of 811 (2.1%) *KRAS/BRAF* WT tumours compared with one of 421 (0.2%) tumours harbouring a *KRAS* or *BRAF* mutation (*p* = 0.01). In the stage IV tumours, strong HER2 staining was present in 24 of 466 (5.2%) WT cases, compared with 5 of 525 (1.0%) of those with a mutation (Fisher's exact test, *p <* 0.0001). This difference was observed in each of the stage IV trial datasets [FOCUS, 14 of 259 (5.4%) versus 4 of 284 (1.4%); PICCOLO, 10 of 207 (4.8%) versus 1 of 241 (0.4%)].

### HER2 and recurrence rate, PFS and OS analysis

**Figure 3:**
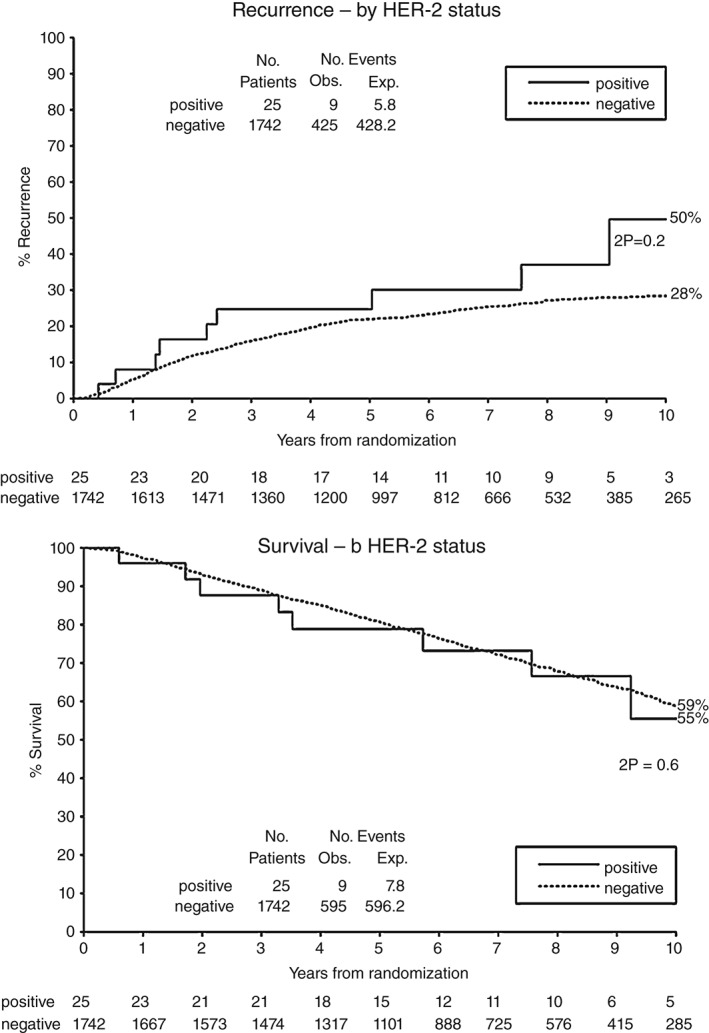
Kaplan–Meier estimates of recurrence and survival of patients in the QUASAR trial, with tumours showing overexpression or no expression of HER2 protein

Clinical data was available in 1767 QUASAR cases. There were no significant correlations between membranous HER2 overexpression and either recurrence or overall survival (OS) (Figure 3). There appears to be a greater difference in recurrence between those cases with HER2 overexpression and those showing no overexpression, although this is not significant in the group receiving chemotherapy. The numbers of recurrences in the treated group are similar to the number of recurrences in the entire trial, and this is reflected in the relative *p* values.

Associations of HER2 expression with OS and progression‐free survival (PFS) were first assessed in the two stage IV trial datasets separately. In neither trial was there a significant association. In FOCUS, comparing patients with HER2 strongly positive tumours (n = 18) versus those with no visible membrane staining (n = 826) gave a hazard ratio (HR) for OS of 0.81 [95% confidence interval (CI) 0.50, 1.32; the HR for PFS was 0.68, CI 0.42, 1.12]. Corresponding estimates from the PICCOLO trial (n = 11 versus n = 434) were 0.98, 95% CI 0.54, 1.78 for OS and 0.80, 95% CI 0.44, 1.46 for PFS.

**Figure 4:**
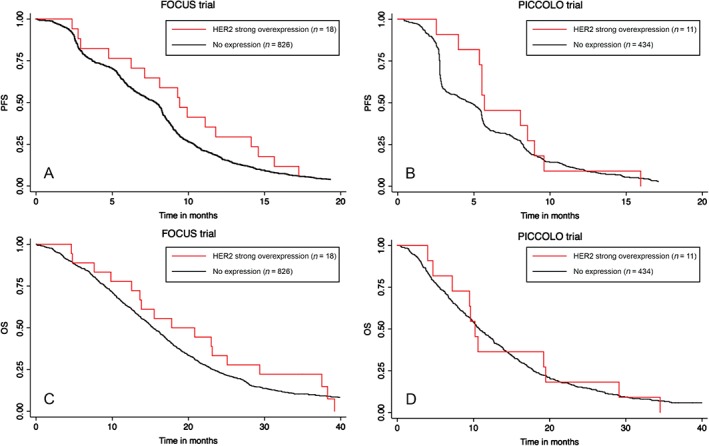
Kaplan–Meier estimates of progression‐free survival (PFS) and overall survival (OS) among patients in FOCUS (A and C, respectively) and PICCOLO (B and D, respectively), with tumours showing overexpression or no expression of HER2 protein

Combining the two datasets, there remains no evidence for an effect on outcome [HR for OS, 0.87, 95% CI 0.60, 1.27; p = 0.48; HR for PFS, 0.73, 95% CI 0.50, 1.07; p = 0.11], with no evidence for heterogeneity between the studies in either analysis (Figure 4). Restricting these survival analyses to patients with KRAS WT tumours, very similar results were obtained [HR for OS from meta‐analysis of the two trials, 0.99, 95% CI 0.65, 1.51; p = 0.95; HR for PFS, 0.83, 95% CI 0.54, 1.28; p = 0.40], with no evidence for heterogeneity between studies.

## Discussion

It has recently been demonstrated in aCRC that, in addition to *KRAS* mutation status, mutations in other proteins downstream of EGFR, including NRAS and possibly also BRAF and PIK3CA, contribute to resistance to anti‐EGFR therapies. Data from trials such as PICCOLO 21 and PRIME 24 have provided strong supporting evidence for this. However, even tumours which are wild‐type for all these genes have a relatively high rate of drug‐resistance.

HER2 has the ability to form heterodimers with EGFR and HER3, and to activate the MAPK and AKT pathways. Overexpression of HER2 is therefore a potential mechanism for circumventing EGFR blockade, making it a potential biomarker of resistance to anti‐EGFR therapy and a potential selection criterion for novel HER2‐ or HER3‐targeted treatments. Bertotti *et al* 25 provided early evidence using HER2‐amplified tumour xenografts, which demonstrated resistance to cetuximab. Accurate characterization of HER2 expression in large patient populations is an important first step to its development as a biomarker in aCRC.

In this study we assessed HER2 expression by immunohistochemistry, identifying a subset of CRC with strong overexpression of membranous HER2. Overall, we observed this in just 25 of 1914 (1.3%) stage II–III tumours and 29 of 1342 (2.2%) of stage IV tumours, which, although a small proportion, still represents a sizeable number of patients in this common disease group. This low level of expression is almost identical to that reported by Lee *et al* 14 when reporting on HER2 overexpression in 94 consecutive stage IV CRCs. We found that 20 of 24 (83.3%) stage II–III patients and 27 of 28 (96.4%) stage IV patients with strong IHC staining also showed *HER2* amplification by FISH.

Several previous studies have assessed HER2 using both IHC and FISH analyses, and also found that gene amplification is a consistent finding in tumours with strong (3+) membranous staining by IHC 9, 11, 15. These studies also identified cases with moderate/weak (2+) staining and equivocal amplification by FISH. However, of the 1342 stage IV patients, we found no cases falling into this intermediate staining category. We identified 53 tumours with visible but weak (1+) staining; 10 of these tumours, selected at random, underwent *HER2* FISH analysis, with none demonstrating gene amplification. These results suggest that gene amplification is associated with strong membranous overexpression of HER2, and that IHC is a reliable technique for assessing HER2 in CRC. It remains unclear whether the HER2 status of these is representative of that of any corresponding metastases. Lee *et al* 14 addressed this very question and determined that in almost 15% of tumours, there was a lack of concordance between the HER2 status in the primary tumour and associated metastases. This may need to be taken into account if future clinical trials are realised.

In order to confirm the amplification of the *HER2* gene seen in the FISH analysis, 47 cases with strong membranous overexpression of HER2 were analysed using the Affymetrix OncoScan FFPE Assay Kit: 43 of 47 (91.4%) showed full or partial amplification of *HER2*, suggesting that the OncoScan method is an alternative way of assessing copy number variation; only four of 47 cases (8.5%) showed no amplification of *HER2*, including one case where analysis by FISH failed and one case where FISH also showed no amplification. We were also able to access OncoScan data on 159 PICCOLO samples, which were negative for HER2 protein expression. None of these tumours demonstrated HER2 amplification. We feel this further evidences the strong correlation between protein expression and amplification, and is confirmation that the negative tumours are not in fact ‘false negatives’. These results suggest that HER2 expression is increased in a small subset of intermediate and aCRC, and that these cases may benefit from anti‐HER2 therapies such as trastuzumab and pertuzumab.

Seventeen of 47 cases (36.2%) showed co‐amplification of *HER2* and *TOP2A*. These results support existing reports of *HER2/TOP2A* co‐amplification in the range 21–42% 26, 27, 28.

In all disease stages, but particularly in stage IV, we observed a significant association of HER2 overexpression with *KRAS/BRAF* WT tumours (*p <* 0.0001). This is consistent with the hypothesis that *HER2* amplification may represent an alternative driver to MEK–AKT pathway activation in tumours without an activating mutation of a downstream oncogene. One might predict that these HER2‐amplified tumours would be resistant to cetuximab or panitumumab therapy, and that they may respond to targeted treatment against HER2, or a combination of therapies targeting HER2, EGFR and possibly also HER3. Such combinations have been reported to inhibit growth in cetuximab‐resistant cell lines 25, 29. A recent clinical study reported *HER2* amplification in seven of 170 (4%) *KRAS* WT patients receiving cetuximab or panitumumab, and these patients had inferior PFS and OS compared to those without *HER2* amplification; however, the small sample size limits interpretation of this result 30.

Recently presented data on the HERACLES trial, a phase II trial in *HER2*‐amplified, *KRAS* exon 2 WT mCRC patients of trastuzumab and lapatinib, achieved its primary endpoint of an objective response (OR) in > 30% of patients. This trial provided the first real evidence that patients with *HER2* amplification may receive benefit from dual anti‐HER2 treatments, and certainly points to the incorporation of such therapies earlier in the treatment of mCRC patients 31. To ensure that the correct patients were enrolled into the HERACLES trial, work was carried out by Valtorta *et al* 32 to develop criteria to accurately define HER2 positivity in colorectal tumours. According to these criteria, HER2 positivity was defined as the presence of intense, homogeneous membranous staining in at least 50% of tumour cells, and these tumours also showed *HER2* amplification by FISH. The study also reported a rate of *HER2* amplification in *KRAS* WT tumours of just over 5%, consistent with our findings of 5.2%. This provides additional evidence that our negative tumours are indeed true negatives.

In the QUASAR trial, we did not find a significant correlation between HER2 expression and recurrence or survival. Furthermore, we did not find an association between HER2 expression and PFS or OS in the stage IV trials. Due to the rarity of HER2 overexpression, the power to detect a modest effect on outcome is low, but we can exclude a strong effect. This agrees with two previous studies, which failed to detect a prognostic biomarker effect for HER2 10, 13.

One area we have not investigated in this study is the presence of activating mutations in the *HER2* gene. A recent functional analysis by Kloth *et al* 33 revealed a differential response to HER2‐targeted therapies in *HER2*‐mutant MSI CRC cell lines. A similar response was also shown by Kavuri *et al* 34, where treatment of *HER2*‐mut CRC cell‐lines with a single HER2‐targeted agent produced delayed tumour growth and treatment with dual agents (anti‐HER2 therapy plus a tyrosine kinase inhibitor) produced tumour regression. Bertotti *et al* 35 recently carried out exome sequence and copy number analyses of both patient‐derived xenografts and patient tumours, to investigate the effects of mutations in *ERBB2*, *EGFR*, *FGFR1*, *PDGFRA* and *MAP2K1* in relation to resistance to anti‐EGFR therapies. Point mutations were detected in both the kinase domain and the ectodomain of *HER2*, and these correlated with cetuximab resistance. This provides early evidence of a role for somatic mutations in the activation of HER2 signalling, resulting in anti‐EGFR therapy resistance. With the advent of next‐generation sequencing, this is one area which ought to be investigated further, with the aim of defining a *HER2*‐mutant cohort who may gain benefit from HER2‐targeted therapies.

In conclusion, we have identified, within these large clinical trial cohorts, a small subset of patients with tumours overexpressing membranous HER2 and having amplification of the *HER2* gene confirmed by FISH and microarray studies. HER2 overexpression is not a prognostic biomarker; however, it is strongly associated with *RAS/RAF* WT status and *TOP2A* amplification. This raises the hypothesis that *HER2* amplification is an alternative driver of MEK–AKT pathway activation in CRC. We propose that HER2 should now be assessed as a putative biomarker of resistance to anti‐EGFR therapy in *RAS/RAF* WT patients, as a potential predictive biomarker for HER2‐targeted therapy and, if further studies confirm that *TOP2A* amplification is associated with anthracycline sensitivity, it might be worth screening for HER2 overexpression to triage for its presence in this disease.

## Author contributions

SDR, KS, JB, MS and PQ conceived and designed the study; SDR, KS, PC, DC, GH, MT, JMF, AO and KGS carried out the data collection and assembly; data analysis and interpretation were performed by SDR, KS, PC, JB, HW, JMF, AO, KGS, SRB, MJ, DK, KH, RG and PQ. All authors were involved in the writing of the manuscript and also approving the final submitted version.
